# Resolution criteria in double-slit microscopic imaging experiments

**DOI:** 10.1038/srep33764

**Published:** 2016-09-19

**Authors:** Shangting You, Cuifang Kuang, Baile Zhang

**Affiliations:** 1 Division of Physics and Applied Physics, School of Physical and Mathematical Sciences, Nanyang Technological University, 637371, Singapore; 2 State Key Laboratory of Modern Optical Instrumentation, Zhejiang University, Hangzhou 310027, China; 3 Centre for Disruptive Photonic Technologies, Nanyang Technological University, 637371, Singapore

## Abstract

Double-slit imaging is widely used for verifying the resolution of high-resolution and super-resolution microscopies. However, due to the fabrication limits, the slit width is generally non-negligible, which can affect the claimed resolution. In this paper we theoretically calculate the electromagnetic field distribution inside and near the metallic double slit using waveguide mode expansion method, and acquire the far-field image by vectorial Fourier optics. We find that the slit width has minimal influence when the illuminating light is polarized parallel to the slits. In this case, the claimed resolution should be based on the center-to-center distance of the double-slit.

With the development of high-resolution and super-resolution microscopies, the resolution of optical microscopy can reach the sub-wavelength scale. In order to verify the resolving capability of a proposed microscope, it is very common to fabricate a sub-wavelength metallic double-slit sample to carry out imaging experiment^1^,^2^,^3^,^4^,^5^,^6^,^7^,^8^,^9^. However, the effects from the slit width and the polarization of the illuminating light were generally neglected. This also raised inconsistency in the claimed resolution: the center-to-center distance of the double-slit was claimed to be the resolution in some researches^1^,^2^,^3^,^4^, while the side-to-side distance of the double-slit, in contrast, was adopted as the resolution in other researches^5^,^6^,^7^,^8^. In view of the fact that it is impossible to fabricate an infinitely narrow slit, it is worthwhile to verify the influence of the slit width, and find out a more consistent criteria where the resolution experiments can be insensitive to the slit width.

We theoretically investigate how the polarization of the illuminating light and the slit width will affect the imaging result. In traditional diffraction theory, the intensity distribution at the output port of a slit illuminated by a uniform plane wave is still considered as uniform. However, this approximation is not applicable for sub-wavelength structure. In this paper, we use waveguide mode expansion method to rigorously calculate the electric field inside and near the double-slit, and acquire the far-field image by vectorial Fourier optics. After the effect of slit width is revealed, we propose a criteria for resolution verification that can provide more consistent results in double-slit microscopic imaging experiments.

## Results

**Figure 1:**
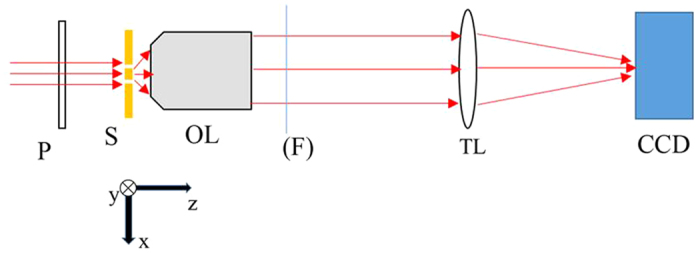
System setup. P, polarizer; S, double-slit sample; OL, objective lens; (F), Fourier spectrum plane; TL, tube lens; CCD, charge-coupled device camera.

The imaging system model is shown in Fig. 1. A 632.8 nm incident plane wave with its polarization controlled by a polarizer illuminates the double-slit sample. The slits extend in the y-direction. We start with a scenario where the two slits can be well resolved in one polarization, but cannot for the other polarization. The slit width is 275 nm, the center-to-center distance of the two slits is 550 nm, and the sample’s thickness is 50 nm. The slit length is much greater than the width, and we regard the slits as infinitely long along the y direction. The diffracted light is collected by an objective lens whose numerical aperture (NA) is 0.9. The objective lens and the tube lens form a 4f system, and the Fourier spectrum plane is at the common focal plane of the two lenses. A CCD camera detects the image.

In order to rigorously solve the electric field distribution inside and near the double-slit, we first expand the electric field inside the metallic slits into waveguide modes. The metal is regarded as perfect electric conductor.


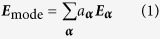


Here, ***α*** = (*α*_1_, *α*_2_, *α*_3_, *α*_4_) is a four dimensional subscript, where *α*_1_ indicates which slit the mode field is located in, *α*_2_ indicates TE or TM mode, *α*_3_ indicates the mode order, and *α*_4_ indicated the forward or backward propagating direction. ***E***_***α***_ is the electric field of mode ***α*** inside the waveguide. The scattered field outside the double-slit can be expressed as


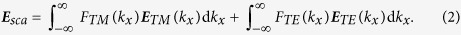


Here, ***E***_*TE*_ is the TE-polarized plane wave outside the slits, and ***E***_*TM*_ is the TM-polarized plane wave outside the slits. *F*_*TM*_ and *F*_*TE*_ are the Fourier spectrum of TM-polarized and TE-polarized electric field, respectively. And *k*_*x*_ is the x-component of the wave vector. Because the slits are assumed to be infinitely long, the diffracted field will not propagate in the direction where *k*_*y*_ ≠ 0, thus there is an assumption in Eq. (2) that *k*_*y*_ = 0. It is easy to acquire the boundary conditions at the input port and output port of the double-slit.


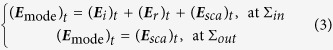



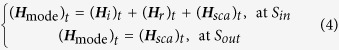


Here, ***E***_*i*_ is the incident field and ***E***_*r*_ is the reflected field when there is no slit on the metallic board. Subscript t represents the tangential component. Σ_*in*_ and Σ_*out*_ are the whole planes of the double-slit structure’s input port and output port. *S*_*in*_ and *S*_*out*_ are the range of the slits in the planes of the input port and the output port.

According to the waveguide mode expansion method of ref. 10, since all the waveguide modes are orthogonal, the boundary condition equations can be projected to every waveguide mode using overlap integral, generating enough linearly independent equations to solve all the *a*_***α***_. Thus we acquire the electric field inside the double-slit. Then we can acquire the electric field outside the double-slit by projecting the electric field inside the double-slit to ***E***_*TE*_(*k*_*x*_) or ***E***_*TM*_(*k*_*x*_) and matching the boundary conditions.

**Figure 2:**
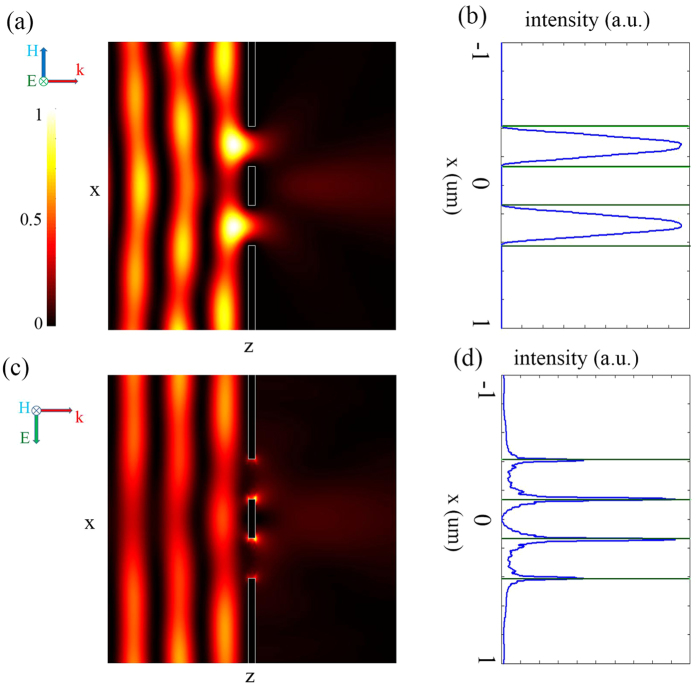
Light intensity distribution near the double-slit. When the incident light is y-polarized: (**a**) intensity distribution in x-z plane and (**b**) intensity profile at the output port of the double-slit. When the incident light is x-polarized: (**c**) intensity distribution in x-z plane and (**d**) intensity profile at the output port of the double-slit. The green lines in (**b,d**) indicate the range of the slits.

In our numerical calculation, we make the highest waveguide mode order to be 10, and make the range of *k*_*x*_ to be [−50*k*_0_, 50*k*_0_], where *k*_0_ is the wave number of the incident light. The calculation has been verified with Comsol simulation to ensure the validity of this method. The calculation result is shown in Fig. 2.

When the incident light is y-polarized, only TE modes can exist in the slits, and TE1 is dominant. The diffracted field is TE-polarized, i.e., only y-polarized electric field component exists in the free space behind the double-slit. Figure 2(a) is the distribution of |*E*|^2^ in the x-z cross section. Figure 2(b) is the light intensity profile at the output port of the double-slit. It is clear that most energy is concentrated in the middle part of each slit.

When the incident light is x-polarized, only TM modes can exist in the slits, and TM0 is dominant. The diffracted field is TM-polarized, i.e., both x-polarized and z-polarized electric field components exist in the free space behind the double-slit. Figure 2(c) is the distribution of |*E*|^2^ in the x-z cross section. Figure 2(d) is the light intensity profile at the output port of the double-slit. It is clear that most energy is concentrated at the edges of the slits. Moreover, the electric field is nonzero out of the range of slits.

Note that the profiles in Fig. 2(b,d) are the normalized intensity, which does not mean the total transmitted energy of one case is greater than the other. Generally, if the slit width is less than half of a wavelength, all TE modes will be cut off, while the TM0 mode will not. Thus, the y-polarized light will exponentially decay in the slits, resulting in a very low transmittance.

Next, we use vectorial Fourier optics^11^,^12^ to calculate the image received by the CCD. The high NA objective lens has two effects. For one thing, it serves as a low-pass filter which blocks out high frequency components. For another, it changes the polarization state. Taking these two effects into consideration, we can obtain the vectorial spectrum in the Fourier spectrum plane with Eqs (5, 6, 7).


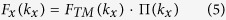



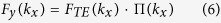



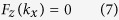


Here, Π(*k*_*x*_) is a window function related to the NA of the objective lens. Note that in obtaining Eqs (5, 6, 7), the condition that, when *k*_*y*_ ≠ 0, *F*_*TE*_ = *F*_*TM*_ = 0, is utilized. If this condition is not satisfied, (e.g. finitely long double-slit), Eqs (5, 6, 7) need to be further modified.

The tube lens transform this spectrum into image on the CCD. Equation (8) gives the image, where **F** represents the Fourier transform.


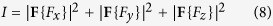


**Figure 3:**
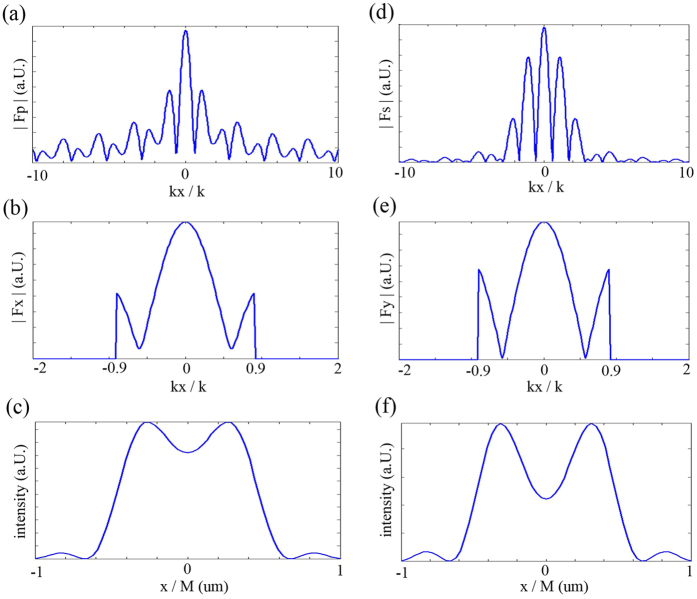
Vectorial analysis of microscope imaging. When the incident light is x-polarized: (**a**) normalized strength of TM-polarized spectrum at the output port of the double-slit, (**b**) normalized strength of x-polarized spectrum at the Fourier spectrum plane, (**c**) normalized intensity of the image. When the incident light is y-polarized: (**d**) normalized strength of TE-polarized spectrum at the output port of the double-slit, (**e**) normalized strength of y-polarized spectrum at the Fourier spectrum plane, (**f**) normalized intensity of the image. M is the magnification of the system.

Figure 3 shows the results at different planes in the imaging process. When the incident light is x-polarized, the scattered field is totally TM-polarized. The spectrum *F*_*TM*_(*k*_*x*_) at the output port is plotted in Fig. 3(a). The objective lens collects the scattered light and the spectrum is formed on the Fourier spectrum plane. In this plane, only x-polarized component exists, as plotted in Fig. 3(b). The tube lens converts this spectrum into an image as shown in Fig. 3(c).

When the incident light is y-polarized, the scattered field is totally TE-polarized. The spectrum *F*_*TE*_(*k*_*x*_) is plotted in Fig. 3(d). The objective lens collects the scattered light and the spectrum is formed on the Fourier spectrum plane. In this plane, only y-polarized component exists, as plotted in Fig. 3(e). The tube lens converts this spectrum into image as shown in Fig. 3(f). Obviously, when the incident light is y-polarized, the two peaks of the image can be discerned more clearly. Therefore, the polarization of illuminating light indeed has non-negligible influence on the resolution.

Then we vary the spacing between two slits and their width. In order to evaluate the resolution of the image, we introduce a ratio *I*_*pit*_/*I*_max_, where *I*_*pit*_ is the intensity at the pit between the two peaks in the image, and *I*_max_ is the maximum intensity in the image. Hence, the smaller this ratio is, the more likely the double-slit image can be resolved.

**Figure 4:**
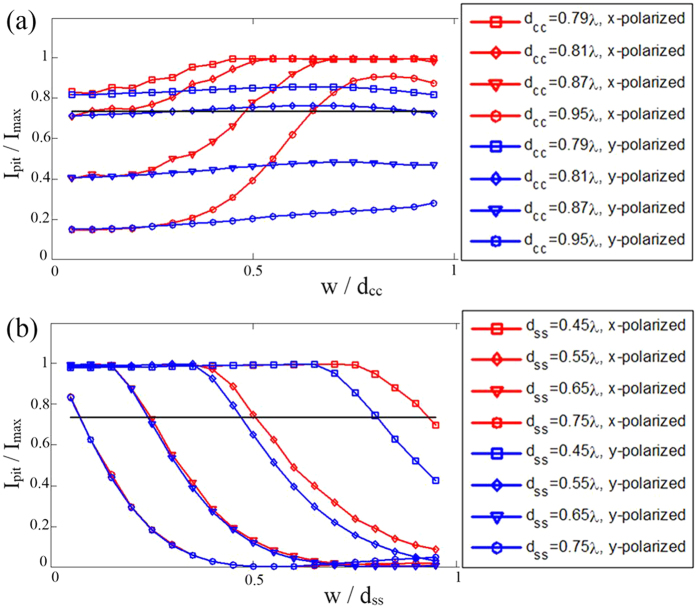
Effect of slit spacing, slit width and polarization. (**a**) Relation between pit intensity and slit width when the center-to-center distance is fixed. (**b**) Relation between pit intensity and slit width when the side-to-side distance is fixed. Horizontal black lines is the critical condition when the two slits can just be discerned.

In some researches, the resolution is claimed based on the center-to-center distance of the double-slit. Here we choose a fixed center-to-center distance from the following values: *d*_*cc*_ = 0.79*λ, d*_*cc*_ = 0.81*λ, d*_*cc*_ = 0.87*λ* and *d*_*cc*_ = 0.95*λ*, and observe the change of *I*_*pit*_/*I*_max_ when the slit width *w* varies from 0.05*d*_*cc*_ to 0.95*d*_*cc*_. The result is shown in Fig. 4(a). It can be seen that when the incident light is y-polarized, *I*_*pit*_/*I*_max_ is insensitive to *w*/*d*_*cc*_; on the contrary, when the incident light is x-polarized, this ratio is very sensitive to slit width.

While in some other researches, the resolution is claimed based on the side-to-side distance of the double-slit, which is *d*_*ss*_ = *d*_*cc*_ − *w*. Here we choose a fixed side-to-side distance from the following values: *d*_*ss*_ = 0.45*λ, d*_*ss*_ = 0.55*λ, d*_*ss*_ = 0.65*λ* and *d*_*ss*_ = 0.75*λ*, and observe the change of *I*_*pit*_/*I*_max_ when the slit width *w* varies from 0.05*d*_*ss*_ to 0.95*d*_*ss*_. The result is plotted in Fig. 4(b). It is clear that, for both polarizations, *I*_*pit*_/*I*_max_ is quite sensitive to *w*/*d*_*ss*_.

It is convenient to mark 0.735 as the critical condition when the two slits can just be discerned^13^. Figure 4(a) implies that 0.81λ is the resolution of a NA = 0.9 microscope system. On the other hand, according to Rayleigh’s criteria, the resolution should be 0.61*λ*/*NA* = 0.68*λ*. This contradiction is because the Rayleigh’s criteria is for incoherent systems. Generally, for a coherent system, if the two point sources are in phase, it has worse resolution than an incoherent system^14^. Here the electric field at the output port of the two slits is in phase. Thus we acquire a worse resolution than the classical prediction.

**Figure 5:**
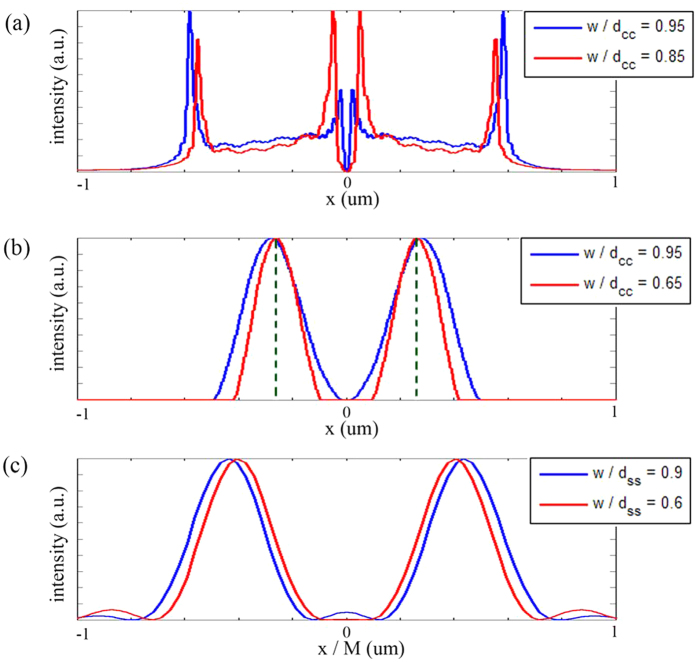
Intensity profile at the output port and of the image. (**a**) Intensity profile at the output port of the double-slit. The incident light is x-polarized and center-to-center distance of the double-slit is 0.95λ. (**b**) Intensity profile at the output port of the double-slit. The incident light is y-polarized and center-to-center distance of the double-slit is 0.81λ. The dashed green lines mark the central position of the slits. (**c**) Intensity profile of the image. The incident light is y-polarized and side-to-side distance of the double-slit is 0.75λ. M is the magnification of the system.

The general tendency of the curves in Fig. 4(a) is ascending, which means if the center-to-center distance is fixed, a larger slit width will result in a worse resolution. However, unusual descent also occurs at the end of some curves, such as the red curve of *d*_*cc*_ = 0.95*λ* and the blue curve of *d*_*cc*_ = 0.81*λ*. The energy distribution accounts for this phenomenon. Figure 5(a) shows the intensity profile at the output port of the double-slit for x-polarized illumination when *d*_*cc*_ = 0.95*λ*, where the slit width is *w* = 0.85*d*_*cc*_ and *w* = 0.95*d*_*cc*_ respectively. The energy distribution is concentrated at the edges of two slits. However, if the two slits are too close, the energy is more concentrated at the outer edges rather than the inner ones. Figure 5(b) shows the intensity profile at the output port of double-slit for y-polarized illumination when *d*_*cc*_ = 0.81*λ*, where the slit width is *w* = 0.65*d*_*cc*_ and *w* = 0.95*d*_*cc*_ respectively. The energy distribution is concentrated at the center of each slit. If the two slits are too close, the peak of energy distribution profile will slightly shift outwards.

The general tendency of the curves in Fig. 4(b) is descending, which means if the side-to-side distance is fixed, a larger slit width will result in a better resolution. However, unusual ascent also occurs at the end of the curves of *d*_*ss*_ = 0.75*λ* for y-polarized illumination. This is because of a small bulge between the two well-separated main peaks. Figure 5(c) shows the intensity profile of the images for the case of y-polarized illumination when *d*_*ss*_ = 0.75*λ*, where the slit width is *w* = 0.6*d*_*ss*_ and *w* = 0.9*d*_*ss*_ respectively. A bulge can be clearly seen between the two well-separated main peaks for *w* = 0.9*d*_*ss*_.

## Discussion

An important observation we get from Fig. (4) is that, when the polarization of incident light is parallel to the extension direction of slits and the center-to-center distance of the double-slit is fixed, the imaging parameter *I*_*pit*_/*I*_max_ is insensitive to the slit width. That is to say, even though a pair of very thin slits are challenging for fabrication, using relatively wide slits in experiment can still well reproduce the result from thin slits, so long as the polarization of incident light is parallel to the direction of slits and the center-to-center distance is taken as the resolution criteria. These conditions can be adopted for more consistent resolution verification in similar high resolution microscopic experiments.

In practical experiment, if possible, the slit width has better to be greater than half a wavelength to enable a propagating TE mode within the slits. Another thing should be noticed is that there will be no TM light induced if the criteria are adopted, hence the proposed criteria is not suitable to some experiments where TM light is necessary.

In conclusion, we rigorously study the scattering and imaging process in a double-slit microscopic imaging experiment and propose a criteria for more consistent resolution verification: The illuminating light polarization should be parallel to the extension direction of the slits, and the verified resolution should be based on the center-to-center distance of the double-slit.

## Additional Information

**How to cite this article**: You, S. *et al*. Resolution criteria in double-slit microscopic imaging experiments. *Sci. Rep.* **6**, 33764; doi: 10.1038/srep33764 (2016).
